# Clinical Characteristics of Pediatric Respiratory Tract Infection and Respiratory Pathogen Isolation During the Coronavirus Disease 2019 Pandemic

**DOI:** 10.3389/fped.2021.759213

**Published:** 2022-01-05

**Authors:** Xifeng Tang, Ge Dai, Xiaohui Jiang, Ting Wang, Huiming Sun, Zhengrong Chen, Li Huang, Meijuan Wang, Canhong Zhu, Yongdong Yan, Wujun Jiang

**Affiliations:** ^1^Department of Respiratory Medicine, Children's Hospital of Soochow University, Suzhou, China; ^2^Department of Respiratory Medicine, Children's Hospital of Wujiang District, Children's Hospital of Soochow University, Suzhou, China

**Keywords:** epidemiology, single infection, coinfection, COVID-19 epidemic, comparison

## Abstract

**Objective:** We sought to compare the clinical characteristics of pediatric respiratory tract infection and respiratory pathogen isolations during the coronavirus disease (COVID-19) pandemic to those of cases in 2018 and 2019.

**Methods:** Our study included all children from 28 days to 15 years old with respiratory tract infections who were admitted to the Department of Respiration, in the Children's Hospital of Soochow University, between January 2018 and December 2020. Human rhinovirus (HRV) and human metapneumovirus (hMPV) were detected by reverse transcription polymerase chain reaction (RT-PCR). *Mycoplasma pneumoniae* (MP) and human bocavirus (HBoV) were detected by real-time fluorescence quantitative polymerase chain reaction (qPCR); In parallel, *Mycoplasma pneumoniae* was detected by enzyme-linked immunosorbent assays, and bacteria were detected by culture in blood, bronchoalveolar lavage specimen, and pleural fluid.

**Results:** Compared to 2018 and 2019, the pathogen detection rate was significantly lower in 2020. With regard to infections caused by single pathogens, in 2020, the detection rates of MP were the lowest and those of HRV were the highest when compared to those in 2018 and 2019. Meanwhile, the positive rates of respiratory syncytial virus (RSV) and hMPV reported in 2020 were less than those recorded in 2018 but similar to those recorded in 2019. Also, the 2020 rate of adenovirus (ADV) was lower than that recorded in 2019, but similar to that recorded in 2018. There were no statistical differences in the positive rates of HBoV and PIV III over the 3 years surveyed. Infections in infants were significantly less common in 2020, but no significant difference was found among children aged 1 to 3 years. The detection rate of pathogens in children old than 5 years in 2020 was significantly lower than those recorded in the previous 2 years. Notably, the pathogen detection rates in the first and second quarters of 2020 were similar to those recorded in the previous 2 years; however, the rates were reduced in the third and fourth quarters of 2020. As for co-infections, the positive rate was at its lowest in 2020. In the previous 2 years, viral–MP was the most common type of mixed infection. By contrast, in 2020, viral–viral infections were the most common combination.

**Conclusion:** The pathogen detection rate was significantly reduced in Suzhou City during the COVID-19 pandemic. Public interventions may help to prevent respiratory pathogen infections in children.

## Introduction

Respiratory tract infections are one of the most common causes of hospital admission in pediatrics. Pneumonia is one of the major causes of death in children under 5 years of age, especially in developing countries (1). At the end of 2019, an outbreak of novel coronavirus infections was documented in Wuhan, Hubei Province, China. To prevent the spread of the novel coronavirus, the Suzhou Municipal Government implemented a first-level response on January 26, 2020; it postponed the resumption of work and school, isolated migrants at home, prohibited markets or other forms of gatherings, and also closed theaters, cafes, public bathrooms, and entertainment and leisure venues. In the current study, we compared the clinical characteristics of pediatric respiratory tract infection cases and respiratory pathogen isolations during the coronavirus disease (COVID-19) pandemic of 2020 with those of cases recorded in 2018 and 2019.

## Methods

### Subjects

Children who were admitted to the Department of Respiratory Disease at the Children's Hospital of Soochow University for respiratory infections from January 2018 to December 2020 were selected. The patients included ranged in age from 1 month to 15 years old and their nasopharyngeal secretions or alveolar lavage fluid were collected for analysis. This study was approved by the Medical Ethics Committee of the Children's Hospital of Soochow University.

### Specimen Collection

Nasopharyngeal secretions or alveolar lavage fluid collected within 24 h after admission were analyzed. We performed a bronchoscopy on patients suspected to have airway malformations or those with pulmonary atelectasis, etc. During this process, a suction catheter was inserted ~7 to 9 cm into the nasopharynx, and approximately 2 mL of nasopharyngeal secretions was obtained and sent for analysis within 30 min. The operator extended the bronchoscope from the nasal cavity through the epiglottis to the trachea and employed physiological saline to irrigate more portions of the airway secretions. Collected alveolar lavage fluid was stored in a sterile collector using a negative pressure suction device and sent out for analysis within 30 min. Seven common respiratory viruses, including respiratory syncytial virus (RSV), influenza virus A (IVA), influenza B (IVB), parainfluenza virus (PIV) I, PIV II, PIV III, and adenovirus (ADV), were detected by direct immunofluorescence assay, while human rhinovirus (HRV) and human metapneumovirus (hMPV) were detected by reverse transcription polymerase chain reaction (RT-PCR). The presence of a bacterial pathogen was confirmed by the following: the detection of *Haemophilus influenza, Moraxella catarrhalis*, and other Gram-negative bacteria or *Streptococcus. pneumoniae* or *Streptococcus pyogenes* in blood, bronchoalveolar lavage specimens, or pleural fluid by culture; a significant increase in *Mycoplasma pneumoniae* immunoglobulin (Ig)G; or the presence of IgM antibodies with *Mycoplasma pneumoniae* DNA (2). Specific IgM and IgG antibodies against *Mycoplasma pneumoniae* were detected in the serum samples of patients in the acute phase of and the recovery phase of *Mycoplasma pneumoniae*, respectively, using a commercial enzyme-linked immunosorbent assay (ELISA) kit (Serion ELISA classic M. pneumoniae IgG/IgM, Institute Virion/Serion, Würzburg, Germany) according to the manufacturer's instructions. The test cutoff value was 0.5×mean optical density (OD) of the kit control serum, as indicated in the insert. A positive IgG reaction was defined as a result of > 24 RU/mL. A significant rise in the IgG titer was considered to be a doubling of the OD value above the cutoff, or a sero-conversion in which the primary serum was negative for antibodies and the second serum sample had an OD value at least twice the cutoff corresponding to a three-fold rise in the RU/mL. A positive IgM antibody reaction was defined as > 1.1 S/CO.

### Data Collection

Demographic, clinical, laboratory, and radiological data were collected retrospectively from all children. Nasopharyngeal secretions, bronchoalveolar lavage specimens and pleural fluid were collected. Considering the growth and development characteristics of children in China, this study divided the children according to age into the following groups: infancy (28 days−1 year), early childhood (1–3 years), preschool (3–5 years), and school-age (>5 years). Children aged 3 to 5 years old go to kindergarten in China, and those older than 5 years of age are allowed to go to primary school.

### Statistical Analysis

Statistical analyses were performed using the Statistical Package for the Social Sciences version 26.0 software program (IBM Corporation, Armonk, NY, USA). Data are expressed as numbers with percentages and mean values. Normally distributed continuous variables were compared using a *t*-test, and non-normally distributed variables were analyzed using the Mann–Whitney *U* test. The chi- squared (χ^2^) test or Fisher's exact test was used to analyze categorical data. *Post-hoc* multiple comparisons were performed to determine the origins of significant differences, and the results were adjusted by using the Bonferroni method.

## Results

A total of 1,948 pediatric cases were enrolled in 2018, and the detection rate of at least one pathogen in this group was 43.79% (853 out of 1,948). Among the patients treated in 2018, 523 patients were male, 361 (42.32%) were younger than 1 year old, 233 (27.32%) were 1 or 2 years old, 120 (14.07%) were 3 or 4 years old, and 139 (16.30%) were at least 5 years old. In addition, a total of 1,796 children treated in 2019 were enrolled in this study, with a positive rate of 50.89% (914 out of 1,796) of these patients, 59.19% (541/914) were male, 277 (30.31%) were younger than 1 year old, 212 (23.19%) were 1 or 2 years old, 185 (20.24%) were 3 or 4 years old, and 240 (26.26%) were at least 5 years old. Finally, ~1,559 children treated in 2020 met the study inclusion criteria for enrollment, and 29.83% (465 out of 1,559) of them had at least one respiratory pathogen. In addition, 268 (57.63%) of these patients were male, 157 (33.76%) were younger than 1 year old, 153 (32.90%) were 1 or 2 years old, 90 (19.35%) were 3 or 4 years old, and 65 (13.98%) were at least 5 years old.

Compared to the rates of the previous 2 years, the pathogen-detection rate in 2020 was significantly reduced (χ^2^ = 155.566; *P* < 0.001).

### Single Pathogen Infections

In 2018, the most common pathogen was RSV, with a positive rate of 8.62% (168/ 1,948), followed by MP at 7.91% (154/ 1,948), HRV at 6.26% (122/ 1,948), HBoV at 3.44% (67/ 1,948), hMPV at 3.18% (62/ 1,948), PIV III at 2.31% (45/ 1,948), and ADV at 0.46% (9/ 1,948). In contrast, the most common pathogen in 2019 was MP, with a positive rate of 17.15% (308/ 1,796), while the second most common pathogen was RSV at 6.68% (120/ 1,796), followed by HRV at 4.79% (86/ 1,796), HBoV at 3.19% (59/ 1,796), PIV III at 1.56% (28/ 1,796), hMPV at 1.50% (27/ 1,796), and ADV at 1.0% (18/ 1,796). In 2020, 136 (8.72%) children had HRV, making it the most common pathogen; this was followed by RSV at 6.16% (96/ 1,559), MP at 3.4% (53/ 1,559), HBoV at 2.63% (41/ 1,559), PIV III at 1.86% (29/ 1,559), hMPV at 1.03% (16/ 1,559), and ADV at 0.51% (8/ 1,559) (Table 1).

**Table 1:** Predominant pathogens of single infections over the 3-year study period.

	**MP^a^^b^^c^**	**RS^b^**	**HRV^b^^c^**	**hMPV^a^^b^**	**HBoV^d^**	**ADV^a^^c^**	**PIV III^d^**
2018	154 (7.91)	168 (8.62)	122 (6.26)	62 (3.18)	67 (3.44)	9 (0.46)	45 (2.31)
2019	308 (17.15)	120 (6.68)	86 (4.79)	27 (1.50)	59 (3.29)	19 (1.0)	28 (1.56)
2020	53 (3.40)	96 (6.16)	136 (8.72)	16 (1.03)	41 (2.63)	8 (0.51)	29 (1.86)
χ^2^	191.391	9.109	21.559	23.928	2.025	16.318	2.841
P	<0.001	0.011	<0.001	<0.001	0.363	<0.001	0.242

a *Significant difference was observed in the pathogen-detection rate among children in 2018 and 2019*.

b *Significant difference was observed in the pathogen-detection rate among children in 2018 and 2020*.

c *Significant difference was observed in the pathogen-detection rate among children in 2019 and 2020*.

d *No significant difference was observed in the pathogen-detection rate during the 3-year study period*.

Compared to in the previous 2 years, the positive rate of MP in 2020 was at its lowest while the detectable rate of HRV was at its highest. Both the rates of RSV and hMPV in 2020 were less than those in 2018 but similar to those in 2019. Meanwhile, there was no statistical difference in the positive rates of HBoV and PIV III during the 3- year study period. Moreover, the rate of ADV was lower in 2020 than in 2019 but was similar to that in 2018.

**Figure 1:**
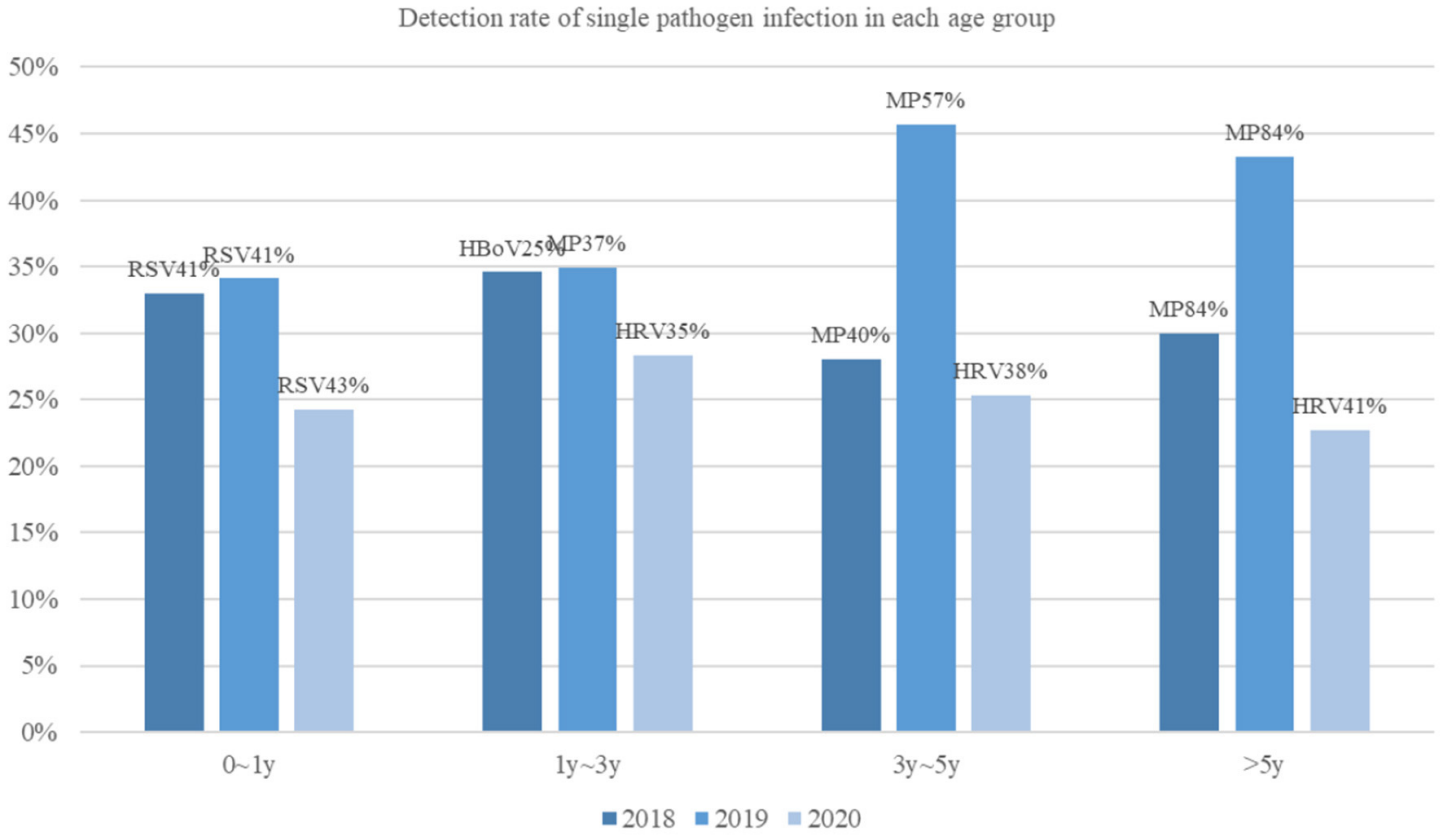
Detection rate of single pathogen infection in each age group.

Infections in both infants and children older than 5 years of age were significantly reduced in 2020 compared to during the previous 2 years. Also, no significant difference was found in the pathogen-detection rate among children aged 1 to 3 years (Figure 1). Overall, the epidemiological of pathogens by age was different. In 2020, the most common pathogen in infants was still RSV; however, children older than 1 year of age, HRV was the most common, instead of MP or HBoV.

**Figure 2:**
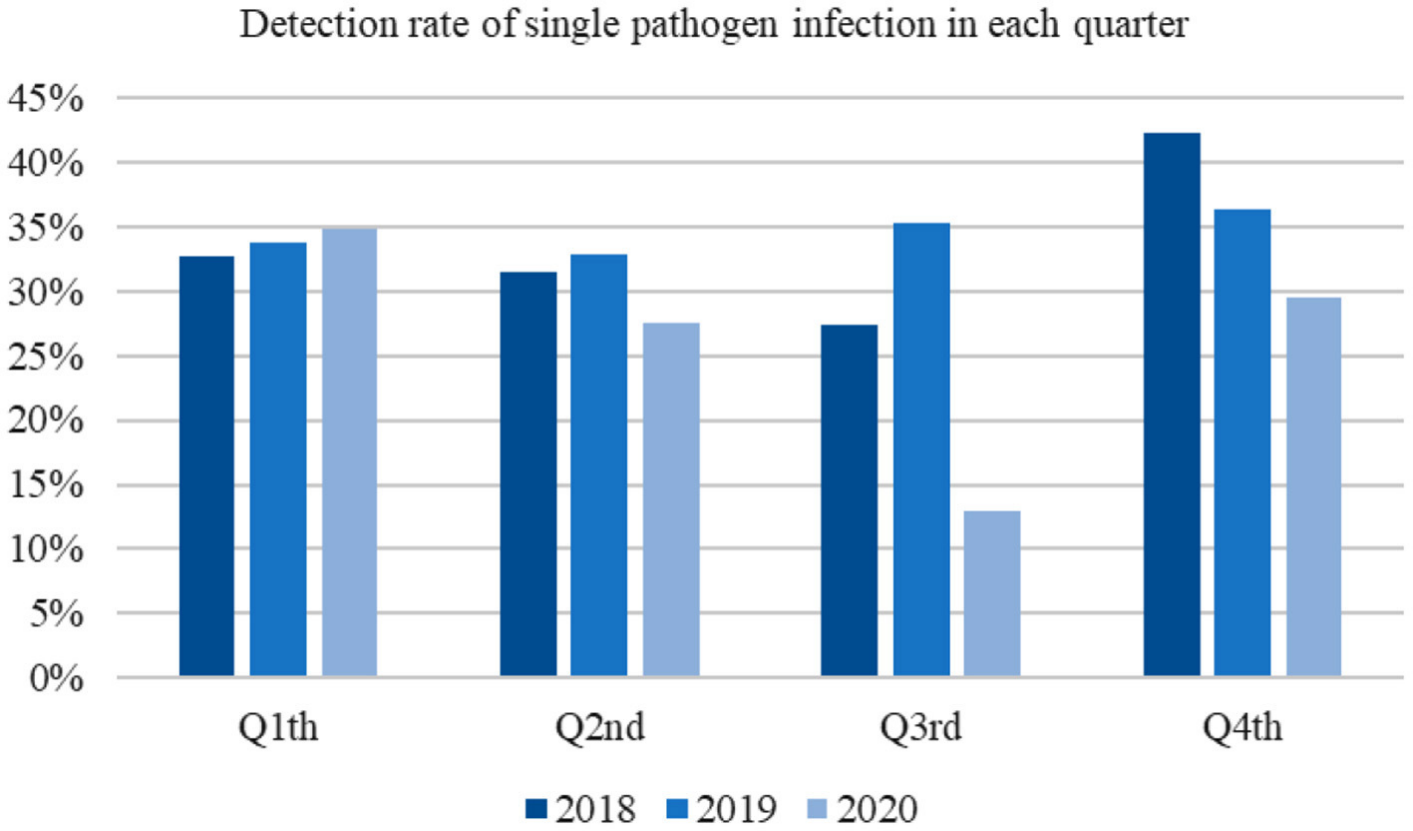
Detection rate of single pathogen infection in each quarter.

The pathogen detection rates in the first and second quarters of 2020 were similar to those recorded in the previous 2 years. In contrast, the rates were reduced in the third and fourth quarters (Figure 2).

### Mixed Infections

Mixed infections were identified in 8.62% (168 out of 1,948) of the patients treated in 2018, and 11.58% (208 out of 1,796) of the patients treated in 2019, respectively; in contrast, the rate of co-infection in 2020 was 3.59% (56 out of 1,559), which was lower than those in 2018 and 2019 (χ^2^ = 72.129, *P* < 0.001).

In 2018, 151 (89.88%) patients had two pathogens, and 17 (10.12%) patients had three pathogens. Mixed viral-bacterial infections were identified in 16(9.52%), mixed viral–MP infections were identified in 88 (52.38 %), mixed viral–viral infections were identified in 58 (34.52 %), and mixed bacterial–MP infections were found in 15 (8.93 %) patients. No bacterial–bacterial mixed infections were detected.

In 2019, 181 (87.02%) patients were found to be infected with two pathogens, while 27 (12.98%) patients were infected with three pathogens. Most of these cases (144 /208, 69.23 %) were mixed viral–MP infections, although mixed viral–viral infections were identified in 38 (18.27%) patients and mixed viral–bacterial infections were identified in 10 (4.81%) patients. Mixed bacterial–MP infections were identified in 35 (16.83 %) patients.

In 2020, 52 (92.86%) patients were infected by two pathogens and four (7.14%) patients were infected with three pathogens, with the most common type of co-infection being viral–viral infections, which were found in 37 of 56 (66.07%) patients. Additionally, mixed viral–MP infections were found in 20 (35.71%) patients, and mixed bacterial–MP infections were detected in five (8.93%) patients.

In 2020, the proportion of viral–viral infections among all cases of mixed infections increased significantly (χ^2^ = 49.115, *P* < 0.001) and the proportion of viral–MP infections among mixed infections was similar to that in 2018 but lower than that in 2019 (*P* = 0.031 and *P* < 0.001). There was no significant difference in the proportion of bacterial–MP infections among mixed infections in the past 3 years (χ^2^ = 6.056; *P* = 0.048).

**Figure 3:**
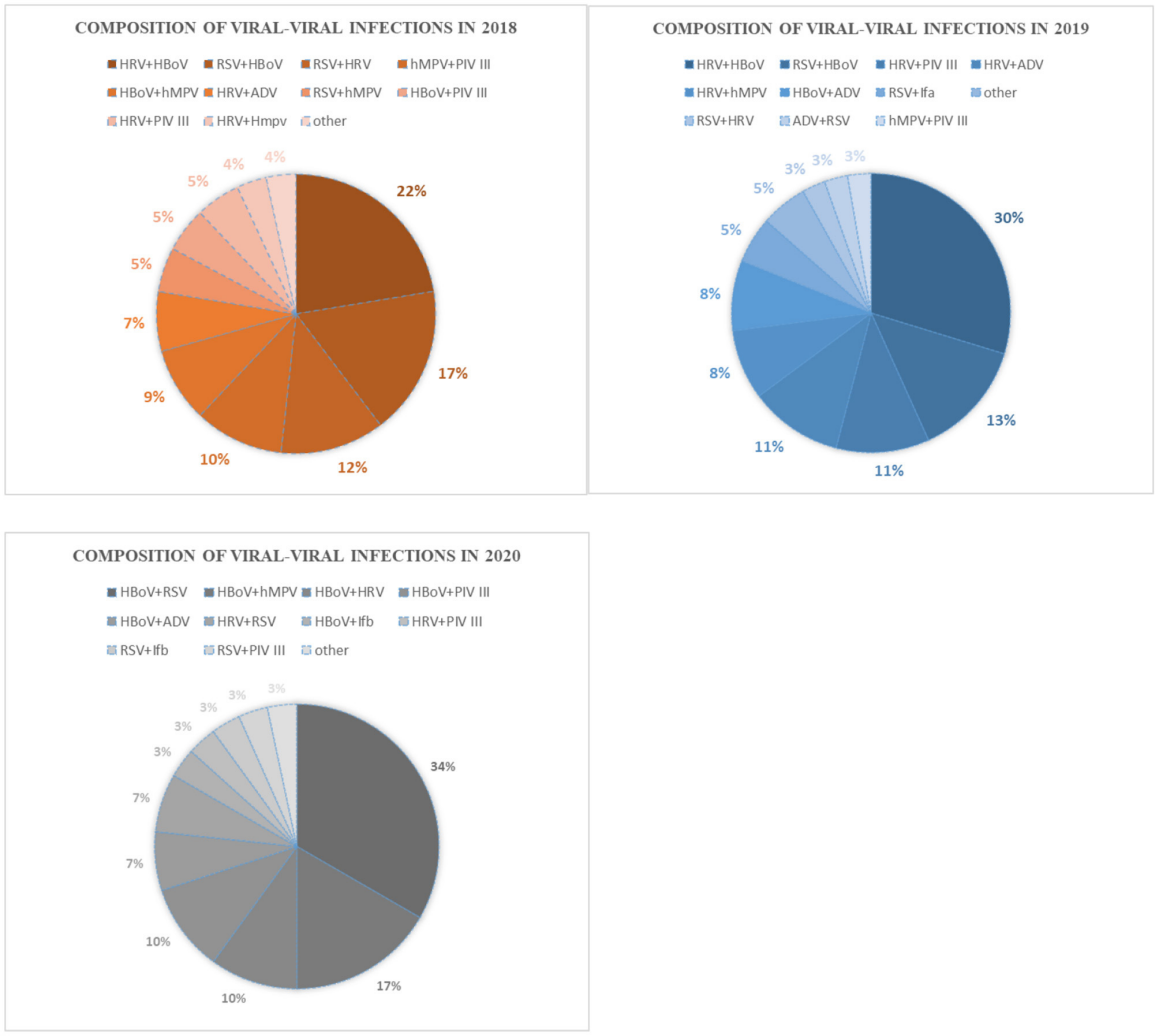
Composition of viral-viral infections in 2018, 2019, and 2020.

**Figure 4:**
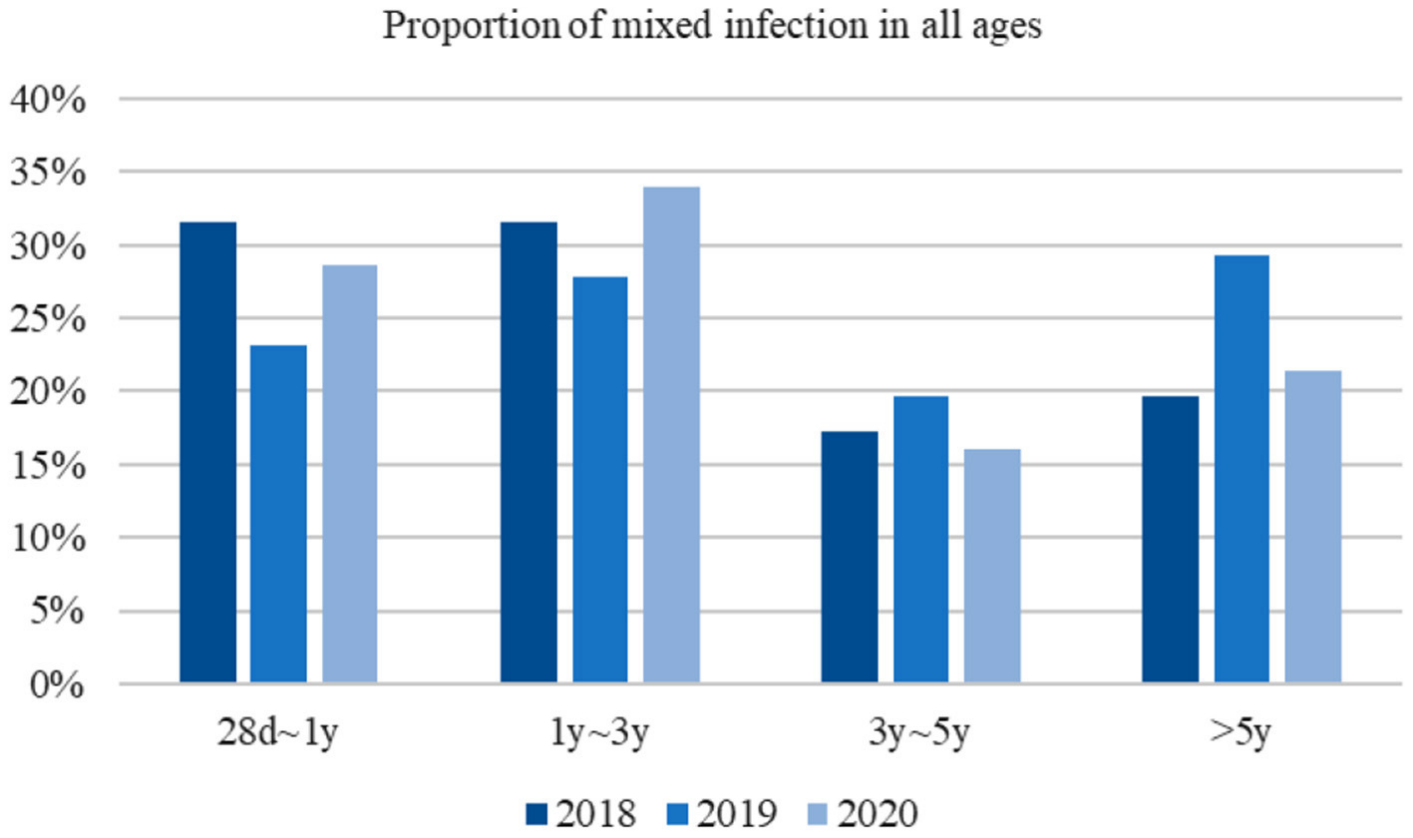
Proportion of mixed infection in all ages.

HRV–HBoV was the most common type of co-infection (22.41%) among viral–viral infections in 2018, followed by RSV–HBoV (17.24%) and RSV–HRV (12.07%). In 2019, the most common type of viral–viral infection was HRV–HBoV (28.94%), followed by RSV–HBoV (13.16%) and HRV–PIV III (10.53%). HBoV–RSV (27.03%) was the most common viral–viral infection in 2020, followed by HBoV–hMPV (13.51%) and HBoV–HRV (8.11%) (Figure 3). There was no significant difference in the proportion of mixed infections over the 3 -year study period according to age group (Figure 4).

### Comparison of Clinical Characteristics of Mixed Infections During the 3-Year Study Period

The demographic and clinical characteristics of patients with co-infections are shown in Table 2. The median age of children was similar, there was no significant difference in the proportion of males, and the average length of admission was neither shortened nor extended between year groups. Moreover, the proportion of children presenting with fever, dyspnea, and wheezing was not significantly different between year groups. Overall, however, the proportion of children presenting with nasal congestion and rhinorrhea was greater in 2020 than in 2018 or 2019. Moreover, the average white cell blood count was lower in 2020 than that in 2018, while the percentage of neutrophils and the C-reactive protein level were similar to those in previous years. There were 28 patients diagnosed with severe pneumonia in 2018, 57 patients diagnosed with such in 2019, and 10 patients diagnosed in 2020. In 2020, the number of cases of severe pneumonia among those with mixed infections was at its lowest. The rate of severe pneumonia in 2019 was higher than that in 2018, but there was no significant difference in the proportion of severe pneumonia in 2020 compared to either 2018 or 2019 (*P* = 0.081 and 0.145).

**Table 2:** Comparison of clinical characteristics of mixed infections over the 3-year study period.

**Characteristics**	**2018**	**2019**	**2020**	***P*-value**
No. of patients	168	208	56	–
Age, months^a^	35	42	33	0.125
Sex, % male	69.00	62.50	58.90	0.217
Length of stay^a^	10.3	8.7	9.2	0.084
Fever, %	67.90	75.00	62.50	0.115
Wheeze, %	44.60	26.40	42.90	0.001
Stuffy nose rhinorrhea, %	37.50	25.00	58.90	<0.001
Dyspnea, %	5.40	3.40	3.60	0.613
Gastrointestinal symptoms, %	27.4	21.20	25	0.368
WBC count, ×10^9^/L^a^	10.8	9.4	9.2	0.017
Percentage of neutrophils^a^	49.00	49.90	48.10	0.836
CRP, mg/L^a^	11.6	10.9	7.5	0.380
Severe pneumonia, %	16.77	27.40	17.86	0.034

a *The mean value was used*.

Data are represented as *n* (%). MP indicates *Mycoplasma pneumoniae*, RSV indicates respiratory syncytial virus, HRV indicates human rhinovirus, hMPV indicates human metapneumovirus, HBoV indicates bocavirus, ADV indicates adenovirus, and PIV III indicates parainfluenza 3.

## Discussion

The ongoing COVID-19 pandemic has impacted people's lives and their travel patterns across the world. The Chinese government has sought to strictly control the spread of severe acute respiratory syndrome coronavirus 2 (SARS-CoV-2) by restricting the gathering and flow of people and by encouraging the wearing of masks, regular handwashing, and frequent disinfection. On January 24, 2020, the Suzhou Municipal Government launched a first-level response; in response, all social activities were stopped and people were told to stay at home. Currently, most people including children old than 3 years of age, still wear surgical masks and maintain social distancing when outside their homes. These precautions may also help prevent the transmission of other viruses, and, as a result, the pathogen-detection rate was significantly reduced in 2020. The most commonly detected pathogens over the 3-year period of our study were RSV, HRV, HBoV, and MP.

### Single Pathogen Infections

The positive rate of MP in 2020 was lower than those reported in the previous 2 years. It is unclear whether this finding is due to the epidemiological characteristics of MP itself or the impact of public interventions. This result is also in agreement with the findings of Zhang et al. (3) who assessed cases of MP infections in Chengdu, China, every month from January 2017 to December 2020, and found that limiting close contact reduced the transmission of MP. It was confirmed that public-intervention measures can effectively reduce MP infection rates. In contrast, our study showed that the detectable rate of HRV was highest in 2020, which is in agreement with the result of a previous study in Japan (4). HRV is one of the most common pathogens capable of causing respiratory infections. Rhinoviruses can be acquired from finger contact with the nasal secretions from an infected individual (5). Although ethanol-containing hand rubs have been widely used during the COVID-19 pandemic, alcohol rubs do not remove rhinovirus from the skin; however, hand-washing with water and soap can remove the virus effectively (6). There were no significant differences between detection of HRV with or without surgical masks, both in respiratory droplets and in aerosols (7). The number of patients infected by other popular respiratory viruses in 2020 such as RSV, hMPV, HBoV, and ADV were less than those in 2018 or 2019; in other words, the competitive inhibitory effect of common respiratory viruses on rhinoviruses was weakened. And it was hard to take strict social distancing for children, especially those returning to school. These may explain the spike in rhinovirus infections in 2020. There were 23 of 404 patients with single pathogen infections diagnosed with severe pneumonia in 2020, 54 of 689 patients diagnosed with such in 2019, and 51 of 646 patients diagnosed in 2018. The number of cases of severe pneumonia among the patients with single pathogen infections in 2020 was at its lowest. This may also explain the increase in the absolute value of HRV infections. Simo et al. (8) discovered a remarkable reduction in HRV prevalence, which was different from our findings. It is worth noting that the observed reduction occurred in the months following the introduction of public interventions and our study analyzed entire period of 2020, including both before and after public interventions. Notably, when such measures began to be eased, the prevalence of HRV increased (8). We must consider whether public interventions will inhibit or delay the spread of respiratory pathogens.

Common respiratory viruses such as RSV, ADV, and influenza virus share a route and means of transmission with SARS-CoV-2. As a result, the measures enacted to control the spread of SARS-CoV-2 also limited that of RSV (9). Zhejiang Province is adjacent to Suzhou, and a study that collected data on respiratory pathogens detected in outpatients from January to April 2020, when the outbreak was at its peak, showed that tests for respiratory virus infections and positive cases of RSV and ADV decreased (10). In our study, the case-positivity rate of RSV was lower than that in 2018 but similar to that reported in 2019, while that of ADV was lower than that in 2019 but similar to that in 2018. The main difference between the two aforementioned studies is that the former compares the number of cases of infection rather than the case-positivity rate.

### Mixed Infections

Most mixed infections in 2018 and 2019 were bacterial–viral cases, which was similar to the findings of a previous study (11).

Meanwhile, the number of co-infections was reduced in 2020. In contrast to during the previous 2 years, the most common type of mixed infection was viral–viral co-infections. In the current study, 26 (46.43%) of 56 patients were infected by HBoV, while in previous studies, some scholars had found that up to 75% of HBoV cases were actually cases of co-infections involving other viruses (12). One study in Europe estimated the rate of HBoV co-infections with other viruses to be 52.4 or 54.1% (13). RSV, ADV, and HRV were more commonly detected concurrently with HBoV than other pathogens. The action mechanism of HBoV and its role in mixed infection cases remain uncertain. We know that HBoV can be detected as many as 6 months in the upper respiratory tract of patients, which may explain the high rate of co-infection with other pathogens (14). It is important to note that not all co-infections are synergistic. HBoV1 can inhibit interferon production *in vitro* and may interfere with rhinovirus-induced immune responses (15).

We found no significant differences in clinical features over the 3-year period of our study, except in the percentage of patients with nasal congestion and rhinorrhea. Our small sample size and increased detection rate of HRV may explain this.

The proportion of severe pneumonia cases among patients with mixed infections in 2020 was similar to those in 2019 and 2018, and most cases included mycoplasma pneumoniae. One study reported that viral co-infection rate in patients with *Mycoplasma pneumoniae* pneumonia to be 56.07 % and mixed ADV was the most common co-presenting pathogen (16). It has also been confirmed that co-infections aggravate disease in children with refractory *Mycoplasma pneumoniae* pneumonia (RMPP) (17).

Our study has some limitations that warrant discussion. Our samples were selected from Suzhou City, which does not fully reflect epidemiological changes due to geographical limitations. Moreover, the relationship between the prevalence of respiratory viruses and public health interventions was not specifically analyzed, and the detailed epidemiological changes of a particular respiratory virus were not investigated. Finally, all of the children enrolled in our study were inpatients as it was not possible to obtain the epidemiology of respiratory viruses in outpatients.

## Conclusion

Public interventions by the government can influence the prevalence of common respiratory viruses. Compared to in 2018 and 2019, overall, the pathogen-detection rate in 2020 was significantly reduced. Notably, in 2020, the detection of MP was significantly reduced, while that of HRV was at its highest. Moreover, viral–viral infections were the most common type of co-infection, and patients were infected with HBoV most frequently. Although we selected patients who were admitted by December 2020, the pandemic continues; therefore, epidemiological changes in respiratory pathogens still require further statistical analysis. Adequate follow-up interventions may prevent respiratory infections in children, and the effectiveness of pandemic and epidemic control may be accurately determined by the epidemiological analysis of a particular virus.

## Data Availability Statement

The raw data supporting the conclusions of this article will be made available by the authors, without undue reservation.

## Ethics Statement

The study was proved by the Medical Ethics Committee of Children's Hospital of Soochow University.

## Author Contributions

All authors have read and approved the manuscript. XT and GD wrote the main manuscript text. YY and WJ conceptualized and designed the study. XJ, TW, and HS collected the clinical data. ZC and LH contributed to the statistical analysis. MW and CZ made contributions to the analysis and interpretation of data.

## Funding

This work was supported by grants from the following projects: The National Natural Science Foundation of China (Grant Nos. 81870006, 81900006, and 81971490); Suzhou Gusu Health Talents Program Stratified Training (key talents) (Grant No. (2020)058). Suzhou Science, Education and Health Science and Technology Project of Youth (Grant No. KJXW2020025). The Science and Technology Program of Suzhou (SYS2020069).

## Conflict of Interest

The authors declare that the research was conducted in the absence of any commercial or financial relationships that could be construed as a potential conflict of interest.

## Publisher's Note

All claims expressed in this article are solely those of the authors and do not necessarily represent those of their affiliated organizations, or those of the publisher, the editors and the reviewers. Any product that may be evaluated in this article, or claim that may be made by its manufacturer, is not guaranteed or endorsed by the publisher.
